# Additive Partitioning of Coral Reef Fish Diversity across Hierarchical Spatial Scales throughout the Caribbean

**DOI:** 10.1371/journal.pone.0078761

**Published:** 2013-10-25

**Authors:** Vanessa Francisco-Ramos, Jesús Ernesto Arias-González

**Affiliations:** Departamento de Recursos del Mar, Centro de Investigación y de Estudios Avanzados del Instituto Politécnico Nacional-Unidad Mérida, Mérida, Yucatán, México; Leibniz Center for Tropical Marine Ecology, Germany

## Abstract

There is an increasing need to examine regional patterns of diversity in coral-reef systems since their biodiversity is declining globally. In this sense, additive partitioning might be useful since it quantifies the contribution of alpha and beta to total diversity across different scales. We applied this approach using an unbalanced design across four hierarchical scales (80 sites, 22 subregions, six ecoregions, and the Caribbean basin). Reef-fish species were compiled from the Reef Environmental Education Foundation (REEF) database and distributions were confirmed with published data. Permutation tests were used to compare observed values to those expected by chance. The primary objective was to identify patterns of reef-fish diversity across multiple spatial scales under different scenarios, examining factors such as fisheries and demographic connectivity. Total diversity at the Caribbean scale was attributed to β-diversity (nearly 62% of the species), with the highest β-diversity at the site scale. $\bar{\alpha }$-diversity was higher than expected by chance in all scenarios and at all studied scales. This suggests that fish assemblages are more homogenous than expected, particularly at the ecoregion scale. Within each ecoregion, diversity was mainly attributed to alpha, except for the Southern ecoregion where there was a greater difference in species among sites. β-components were lower than expected in all ecoregions, indicating that fishes within each ecoregion are a subsample of the same species pool. The scenario involving the effects of fisheries showed a shift in dominance for β-diversity from regions to subregions, with no major changes to the diversity patterns. In contrast, demographic connectivity partially explained the diversity pattern. β-components were low within connectivity regions and higher than expected by chance when comparing between them. Our results highlight the importance of ecoregions as a spatial scale to conserve local and regional coral reef-fish diversity.

## Introduction

Ecologists have been striving to understand the uneven spatial distribution of species [1] and how spatial scales influence biodiversity [2]. There is a growing demand for both large and multiscale analyses to answer crucial questions concerning the origin of diversity and how we might best act in order to maintain it [3]. Traditionally, most attention has been directed toward terrestrial environments, while large-scale diversity patterns in marine ecosystems have received less attention. Recent studies have indicated the need to examine regional patterns of diversity in coral reef systems since their biodiversity is declining on a global scale [4-6]. Such a decline has increased the number of studies on critical groups, including coral reef fishes, which play fundamental ecological roles and are an integral part of coral reefs [6]. 

Coral reef fish communities vary across multiple scales as a function of dispersion, environmental variables and/or inter- and intra-specific relationships [4,7,8]. It is important to understand the spatial scale at which diversity patterns are generated in order to conserve regional and local diversity [9]. Additive partitioning provides a way of exploring diversity distribution over a range of user-defined spatial scales [10,11]. In the additive approach γ-diversity (regional diversity) is partitioned into the sum of the average α-diversity (local diversity) and the β-diversity. Additive association allows diversity measures such as species richness to be hierarchically partitioned because β-diversity at each level is measured in the same units (i.e. species presence). The within-community diversity at a higher spatial level is a combined effect of heterogeneity at various lower levels [12,13]. Furthermore, the null models generated in the partitioning allow comparisons between observed and expected α and β-diversity.

Additive partitioning of diversity has mostly been studied in terrestrial ecosystems using bats [14], birds [15], insects [2,13,16-18], and plants [12,19]. Marine studies applying additive partitioning of species diversity are less common, but include benthic macroinvertebrates [20], stony corals [21,22], and coral reef fish [21,23,24]. Rodríguez-Zaragoza and Arias-González [23] applied additive partitioning to fish diversity at different spatial scales for 11 coral reef sites along 400 km of coast in the Mexican Caribbean. These authors found that at the reef scale, observed α- and inter-habitat β-diversity were higher than expected (from a random distribution), while observed β-diversity among sites was lower than expected. They also observed a small contribution of $\bar{\alpha }$-diversity with the highest β-diversity at the largest spatial scale among reefs. Based on these results and the bioregionalization of the Caribbean Sea by Spalding et al. [25], we addressed the following questions: can we detect changes in the reef fish diversity partition pattern when moving through higher hierarchical spatial scales (sites, subregions, regions, and the Caribbean basin); if so, do the observed relative sizes of partitions reflect more than random sampling? And which spatial scale is associated with the highest β-diversity? We hypothesize that our highest comparable spatial level (ecoregions) will present the highest β-diversity. Diversity in each ecoregion is expected to be relatively different due to reasons related to their biogeography and history. 

Fisheries could also affect the diversity patterns of reef fish [26,27]. The direct effects of fishing depend on the relative impact on common versus rare species [28]. In the Caribbean, the biomass of predatory fishes on coral reefs has been reduced [29]. Fisheries impacts are usually reflected in the size and abundance of target species, although in some cases they can drive species to local extinction (e.g. Nassau grouper) [30]. Therefore, we attempt to detect fishing effects on the diversity-partitioning pattern using the hierarchical spatial scales design, the bioregionalization proposed by Spalding et al. [25] and the fishing target species list of the Western Atlantic-Region 31 [31]. Depending on the spatial distribution of the fishing target species we predict changes in the dominance of the β-diversity components due to fishing. 

Other factors rather than fisheries could affect the spatial distribution of species. Dispersal processes can yield large-scale patterns in species richness but also seem to determine which species are present within local assemblages [7]. Most of reef-fish species are sedentary partly due to their nature but also because the spatial structure resulting from the patchy environment restricts their movement. Most of the connectivity in these species is through the dispersal of larval stages rather than the movements of adult organisms [32]. Therefore, understanding connectivity among coral reef ecosystems is fundamental in order to explain diversity patterns. Cowen et al. [33] studied the likely connectivity of reef-fish species in the Caribbean, using individual-based modeling of larval dispersal in a hydrodynamic field. We also used the connectivity regions proposed by these authors to compare the diversity patterns at different scales. With the partitioning approach we tested the hypothesis that connectivity drives the patterns of α- and β-diversity. We expected low β-diversity within regions and high β-diversity between them. For the different scenarios and scales studied, the results address the importance of the ecoregion as a scale for preserving local and regional fish diversity.

## Material and Methods

### Study sites and database

**Figure 1:**
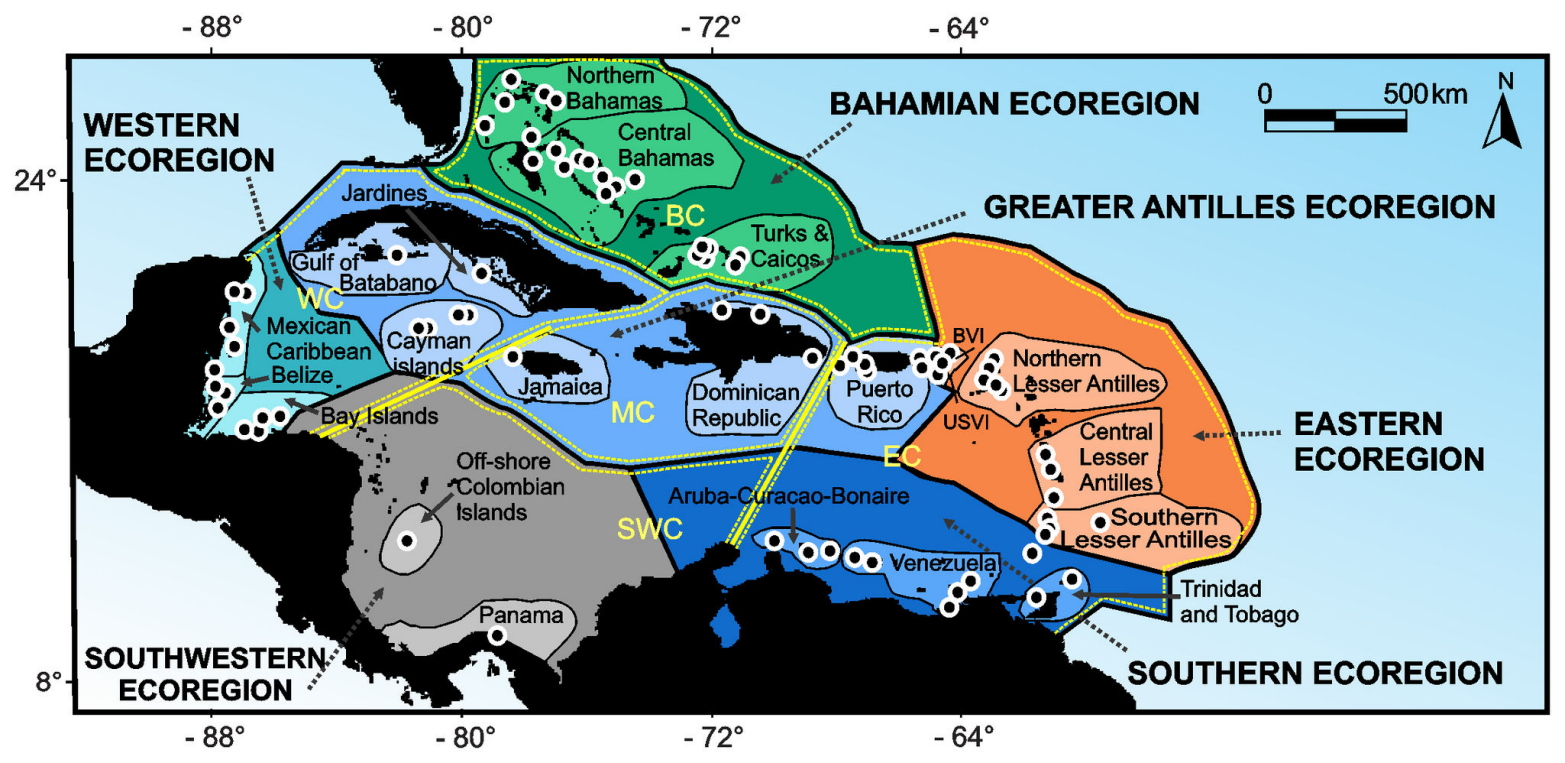
The Caribbean basin with ecoregions, subregions and sites used for the analyses. Bahamian ecoregion: Bahamas, Turks and Caicos; Greater Antilles ecoregion: Cuba, Jamaica, Cayman Islands, Dominican Republic and Puerto Rico; Western Caribbean ecoregion: Mesoamerican reef from Mexico to Honduras; Southwestern ecoregion: Colombian off-shore islands and Panama; Eastern ecoregion: British Virgin Islands (BVI), United States Virgin Islands (USVI) and the Lesser Antilles; and the Southern ecoregion from Aruba to Trinidad and Tobago including Venezuelan off-shore islands. Ecoregions according to Spalding et al. [25]. Subregions within each region are shown by name. Black dots represent the most dived location of each site. Dashed yellow lines delimitate the five connectivity regions according to Cowen et al. [29]. Solid yellow lines represent major biogeographic breaks. See the text for more details on connectivity regions.

We compiled data on the species richness of coral reef-associated fish communities from six ecoregions across the Caribbean (Figure 1). Data were compiled from the online source Reef Environmental Education Foundation (REEF) at http://www.reef.org/, accessed between April and July 2010 [34]. Trained volunteer SCUBA divers using the Roving Diver Technique (RDT) collected data. In the RDT divers swim around a reef site for approximately 45–60 min recording all fish species observed [34]. REEF has two categories of volunteers, beginners and experts; the data collected by each type are kept separate. Volunteers had to take the advanced fish identification exam and conduct a minimum of 35 surveys with the organization to become an expert, which helps to minimize the variation between divers. Only fish censuses by expert divers were used in this study. Schmitt et al. [35] analyzed RDT in comparison with other sampling techniques such as the traditional linear transects and concluded that RDT is a good sampling method for achieving a better representation of the fish community (more species). We used six of the ecoregions of the Tropical Northwestern Atlantic province proposed by Spalding et al. [25]. Currently there is no biogeographic consensus for the regionalization of the Caribbean [36]. Bioregionalization by Spalding et al. [25] was based on taxonomic configurations, influenced by evolutionary history, patterns of dispersal, and isolation. These authors suggested that their classification could be critical for supporting marine biodiversity patterns. Bioregionalization by Spalding et al. [25] includes the Bahamian ecoregion (BE): Bahamas, Turks and Caicos; the Greater Antilles ecoregion (GE): Cuba, Jamaica, Cayman Islands, Dominican Republic and Puerto Rico; the Western Caribbean ecoregion (WE): Mesoamerican reef from Mexico to Honduras. The Southwestern ecoregion (SWE): Colombian off-shore islands and Panama. Eastern ecoregion (EE): British Virgin Islands, United States Virgin Islands and the Lesser Antilles; and the Southern ecoregion (SE) from Aruba to Trinidad and Tobago including Venezuelan off-shore islands. Each region was subdivided into a different number of subregions. The number of subregions was variable within each region. We used a total of 22 subregions across the Caribbean (Figure 1). 

Data included coral reef systems located in 80 sites (77 insular and 3 coastal areas), and consisted of 2670 diving spots with 24,777 expert surveys conducted between 1993 and 2010. We decided to use REEF data because carrying out a scientific campaign of this nature to cover the extension of the Caribbean would be practically and financially impossible. Other studies that used the REEF base include Stallings [37] and Holt et al. [38]. Due to the qualitative nature of species abundance estimations in four categories by RDT divers we only used species presence-absence data. 

We selected study sites with a minimum of 20 surveys based on species accumulation curves (9999 permutations) using EstimateS (v.7) [39], and STATISTICA (v.7.1) [40]. This method has been developed for assessing the completeness of museum and inventory collections; see Jiménez-Valverde and Hortal [41] for details on analyses; we did not make a distinction between protected (e.g. marine reserves, national parks, sanctuaries) or unprotected sites, as long as they achieved the minimum number of surveys. 

The REEF organization has a validation step built in before uploading volunteers’ reports into the data base; however, to reinsure the validity of the species presence in the selected sites we completed a geographical distribution confirmation process based on a wide variety of references including general field guides [42-45], region-specific checklists [46-51], and online information [52,53]. We excluded certain species from the analyses, e.g. hybrids of *Hypoplectrus*, species identified at genus level, and species difficult to identify in the field, such as some belonging to the genus *Elacatinus*, among others. The complete list of all species used in this study can be found in Table S1.

### Richness and Species Composition Overview

In order to compare species richness between the six ecoregions proposed by Spalding et al. [25], the Kruskal-Wallis test was used to determine whether there were significant differences among any of the means (PAST v.2.1) [54]. 

To visualize species composition patterns in assemblages across these ecoregions, we performed non-metric multidimensional scaling (NMDS) based on a Bray-Curtis similarity matrix. We also performed a similarity percentage (SIMPER) routine to assess similarity among subregions and regions based on their species composition (cut off 90%). Data analysis was performed using PRIMER v.6 [55]. 

Permutational multivariate analysis of variance (PERMANOVA; [56]) based on the same similarity matrix was used to test the null hypothesis of no differences among reef fish assemblages at different spatial scales: (1) among subregions within the six ecoregions, and (2) among sites within subregions. Using the hierarchical sampling design, we evaluated the differences at the spatial scale of highest variation in community structure [57]. We used a nested design with 9999 permutations of residuals under a reduced model [58]. Since a significant result for a given factor from PERMANOVA suggests that groups may differ because of their location, their relative dispersion, or both, the Permutational test of multivariate dispersion (PERMDISP) was used to test each of these. PERMDISP were performed to test the factors “subregion” and “site” using a Bray-Curtis similarity matrix for an appropriate comparison [59]. 

To analyze patterns of variation in species composition between the ecoregions we calculated the Russel and Rao similarity coefficient as follows:

$$I_{RR}=\frac{a}{a+b+c+d}$$ (1)

where *a* is the total number of species common to two regions; *b* is the total number of species present in the first region but absent from the second; *c* is the total number of species present in the second region but absent from the first, and *d* is the total number of species absent from both regions but that can be found in the rest of the study area, the Caribbean, in our case.

The Russel and Rao coefficient [60] has an upper boundary of 1 for identical compositions and a lower boundary of 0 when species composition is totally different. We chose the Russel and Rao coefficient over other similarity indexes because of the use of the *d* term (Eq. 1), which allows us to consider the effect of other species from Caribbean coral reefs.

The total number of shared species among regions was calculated and used to generate the percentage of similarity between the six ecoregions. The total number of species present in only one region and only one site (true singletons) was assessed and their distribution according to Froese and Pauly [53] was evaluated to account for cases of endemism.

### Additive partitioning of diversity

Additive partitioning was applied to species richness using an unbalanced design involving four hierarchical spatial scales: sites, sub-regions, regions and the Caribbean basin (Table 1). We performed the hierarchical partitioning analysis excluding the species targeted by the fishery from the entire dataset. For the target species we used the list of commercially important species reported to the Food and Agriculture Organization (FAO) by the countries of the Western Atlantic-Region 31 [31]. Some of the targeted species are not fished specifically for food but are still of commercial value as baitfish (long-line and recreational). The FAO list included 134 species of commercial interest, of which 55 were present in our data. 

**Table 1:** Hierarchical model of species richness.

**Level**	**$\bar{\alpha }$-Diversity**	**β-Diversity**
Caribbean basin	Additive species richness of the Caribbean	Variation in species richness among regions, subregions and sites
Ecoregions (6)	Species richness of each region	Variation in species richness among subregions and sites
Subregions (22)	Species richness of each subregion	Variation in species richness among sites
Sites (80)	Species richness of each site	-

Species richness at each spatial level derived from the sum of α and β-diversities at the next lower level. In parentheses is the total number of samples at each level of analysis.

We performed the diversity partitioning at the Caribbean basin scale and built the primary null model. The grain was always the “site” and the total spatial extent was the Caribbean basin. However, when analyzing the partitioning pattern for each region this became the spatial extent. Total diversity (γ) was partitioned into local $\bar{\alpha }$-diversity (i.e., average diversity within a particular region) and β-diversity (i.e., species variation among selected sites and subregions). The β-diversity component can be calculated as the total diversity minus the average local diversity ($\beta =\gamma -\overset{}{\bar{\alpha }}$). The additive partitioning approach enabled us to directly calculate and compare the contribution of α and β to the total diversity [10]. 

Partitioning was applied at all hierarchical spatial scales, therefore “samples” at one scale are themselves composed of “samples” at a smaller scale. Consequently, γ-diversity was partitioned into the diversity contributed by each scale. At the lower level of analysis, the local average diversity ($\bar{\alpha }$) was calculated among sites. β_1_ is regarded as the average diversity among sites within sub-region; β_2_ is the average diversity among sub-regions within regions; and β_3_ is the average diversity among regions within the Caribbean. These components can be calculated using the following equations:

$${\text{β}}_{\text{1}}\text{=}{\overset{}{\bar{\text{S}}}}_{\text{sr}}\text{-}\overset{}{\bar{\text{α}}}$$ (2)

$$\beta_{2}={\overset{}{\bar{S}}}_{r}-{\overset{}{\bar{S}}}_{sr}$$ (3)

$$\beta_{3}=\gamma -{\overset{}{\bar{S}}}_{r}$$ (4)

where $\bar{S}sr$ and $\bar{S}r$ are the average number of species within a sub-region and a region, respectively. For the Caribbean basin spatial scale the total diversity can be expressed as:

$$\gamma =\overset{}{\bar{\alpha }}+\beta_{1}+\beta_{2}+\beta_{3}$$ (5)

We performed all additive partitioning using the PARTITION v.3 program [61]. We used the reshuffling algorithm of the program to test whether the observed diversity components (α and β) at each spatial scale could have been obtained by a random distribution of individuals among samples at all hierarchical levels. Individual-based randomizations were repeated 999 times. The Individual-based randomization performed by the PARTITION program randomly reassigns each individual presence in the dataset to any given sample level to generate the null model for that dataset. We evaluated statistical significance by determining the proportion of null values that were greater or less than the observed components [61]. Presence-absence data have been previously used to generate null distributions (see Semmens et al. [62]). Obviously when using presence-absence data all species have the same weight. The null distribution used by PARTITION to calculate p-values will not be robust to the sample size and species abundance effects in the same way it would be if abundance data were included. This has to be taken into account when interpreting results from any presence-absence data analysis.

### Fisheries and the diversity partition pattern

In order to detect fishing effects on the diversity partition pattern, we compared the “natural” observed partition distribution that contained all species with the null distribution previously generated for the “unfished” species.

We determined the proportion of null values that were greater or less than the observed components to address statistical differences [61]. We then tested for fishing effects on the diversity partition pattern at the Caribbean basin scale and for each of the ecoregions using the PARTITION v.3 program [61] as described above. 

### Connectivity and the diversity partition pattern

Finally, in order to evaluate whether the demographic connectivity could be driving the α- and β-diversity patterns we performed the partitioning analyses within and between the regions proposed by Cowen et al. [33]. These authors divided the Caribbean into four broadly defined regions of connectivity and a central mixing zone (see yellow lines and major biogeographic breaks in Figure 1). The connectivity regions proposed by Cowen et al. [33] include: the Bahamas and the Turks and Caicos (BC); the Western region (WC), which includes the Mesoamerican Reef, Cayman islands and southern Cuba; the Eastern region (EC), which includes Aruba, Curacao, Bonaire, the Venezuelan coast, Trinidad and Tobago, the Lesser Antilles, British Virgin Islands, US Virgin Islands and Puerto Rico; the region at the periphery of the Colombia-Panama gyre (SWC) and a Central mixing zone (MC) that includes Jamaica and the Hispaniola islands. We reassigned the 80 sites to these five connectivity regions and performed the diversity partition analyses for each region and the pair-wise combination of them using the PARTITION v.3 program [61] as previously described. 

## Results

### Richness and species composition overview

The entire data set consisted of 539 reef fish species belonging to 89 families and 24 taxonomic orders. A complete list of all species is presented in Table S1. Several patterns emerge across the Caribbean in the relationships between local and regional richness of the coral reef fish community. The Southern ecoregion (SE) showed the highest species richness (454), followed by the Eastern ecoregion (422), Western ecoregion (393), Bahamian (379), the Greater Antilles (342), and the Southwestern ecoregion (302). Even though there was no significant inter-region variation in the median number of species (Kruskal-Wallis H = 7.095, p = 0.2136), there was significant variation in the community.

**Figure 2:**
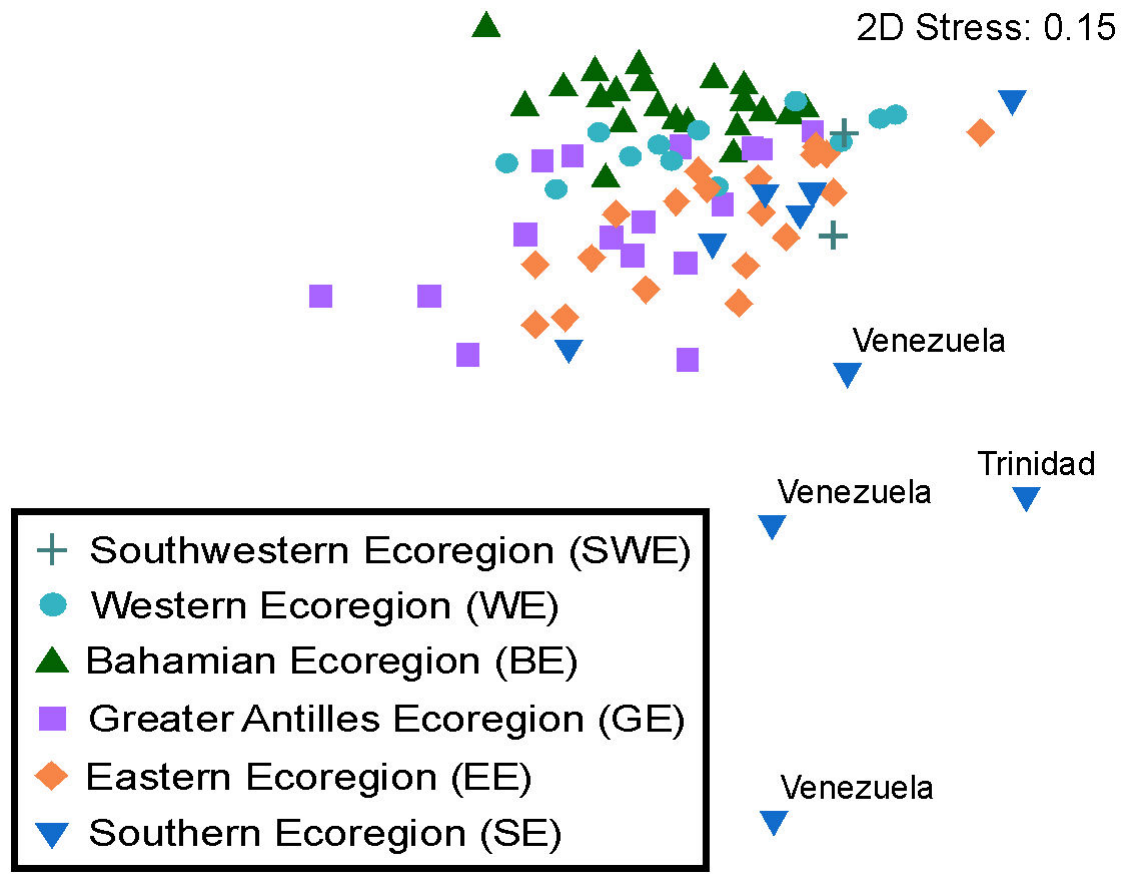
Non-metric multidimensional scaling analysis (NMDS) of fish assemblage structure. Ordination of the 80 sites according to species composition based on a Bray-Curtis similarity matrix. See Figure 1 for details on ecoregions.

Differences in species composition among sites within the Southern ecoregion (SE) were more evident. Compared to the other regions, sites in the Southern ecoregion were more distant from one another (Figure 2). When comparing within ecoregions we found that the Bahamian (BE) showed the highest among-subregions SIMPER similarity percentage, around 78%. The Southern ecoregion (SE) had the lowest among-subregions similarity (67.1%), while the Greater Antilles, Eastern, Western, and Southwestern regions showed similarities of 74.3, 75.8, 76.8 and 77.5%, respectively (Table 2).

**Table 2:** Average similarity percentage (SIMPER), Russel and Rao similarity coefficient (I_RR_) and PERMANOVA pairwise *post* *hoc* test results between the six ecoregions of the Caribbean.

Regions	Average Similarity between regions (%)	I_RR_	PERMANOVA PAIR WISE TEST		
			t	p(perm)	Unique perms
BE-GE	74.11	0.575	2.6011	0.0001	9909
BE-WE	75.04	0.610	2.1713	0.0013	9915
BE-EE	74.41	0.629	2.5775	0.0001	9918
BE-SWE	72.61	0.503	1.8711	0.0043	9930
BE-SE	66.39	0.625	3.0807	0.0001	9946
GE-WE	74.07	0.588	2.1515	0.0029	9946
GE-EE	73.76	0.605	2.4005	0.0006	9918
GE-SWE	72.58	0.495	1.8532	0.0145	9940
GE-SE	67.1	0.601	2.5693	0.0003	9927
WE-EE	72.87	0.655	1.9915	0.0046	9935
WE-SWE	75.46	0.523	1.1728	0.2436	9846
WE-SE	67.45	0.659	2.1448	0.0012	9949
EE-SWE	72.13	0.521	1.4125	0.0984	9934
EE-SE	68.53	0.696	1.8259	0.0049	9934
SWE-SE	68.98	0.544	0.3175	0.3175	9657

p<0.001 differences among groups. Regions abbreviations: Bahamian (BE), Greater Antilles (GE), Eastern ecoregion (EE), Southern ecoregion (SE), Southwestern ecoregion (SWE) and Western ecoregion (WE).

The PERMANOVA showed significant variability at all spatial scales analyzed, with a significant effect of the “ecoregion” grouping (Pseudo-F = 4.3811, p<0.0001). The greatest variability occurred at the smallest spatial scale studied (among sites, within subregions; Pseudo-F = 2.1637, p<0.0001). 

The PERMDISP was also significant, F = 4.3495, p_(perm)_ = 0.0069. The PERMANOVA pairwise *a posteriori* comparison of all ecoregions was significant (p_(perm)_<0.05), except for the comparison between the Western and Southwestern, and the Southern and Southwestern ecoregions (Table 2). 

The Russel and Rao similarity coefficient varied little between ecoregions (Table 2). It was highest between Eastern and Southern ecoregions (0.696), and lowest between the Bahamian and the Southwestern ecoregion (0.503).

We observed 247 fish species in all regions (45.8%) compared to 92 species present in only one region (17.1%). When analyzing the distribution of the species observed in only one region, around 10% of them have been reported to be exclusive to the ecoregion where they were observed (e.g. *Elacatinus atronasum* and *Vomerogobius flavus* only reported for the Bahamian ecoregion). Most of these species has been reported only for the Southern ecoregion, Venezuelan coast, and Trinidad and Tobago (e.g. *Emblemariopsis remirezi*, *Protemblemaria punctata* or *Elacatinus zebrellus*). About 75% of the species we observed in only one of the studied regions have been reported for other regions [53]. Less than 2% of the species observed in just one region are considered endemic (e.g. *Sanopus splendidus* from Cozumel-Mexico, and *Emblemaria diphyodontis* from Cuabagua-Venezuela) [53]. A complete list of these species is presented in Table S2.

Only 63 fish species were present just once in each ecoregion, true singletons. The Eastern (EE) and Southern ecoregions (SE) showed the highest number of singletons. In these two ecoregions we found the highest number of shared species (375 species). However, these regions did not show the highest similarity percentage because both showed the greatest number of species (Table 3). The lowest number of shared species (267) was observed between the Greater Antilles (GE) and the Southwestern ecoregion (SE); while the lowest pairwise similarity percentage was between the Southern (SE) and Southwestern ecoregions (SWE), 63.3%. The Western (WE) and Eastern ecoregions (EE) shared the highest similarity percentage (76.4%), Table 3. 

**Table 3:** Total and percentage of shared coral-reef fish species between six ecoregions of the Caribbean.

	Bahamian	Greater Antilles	Western	Eastern	Southwestern	Southern
Bahamian	379 (100%)					
Greater Antilles	310 (75.43%)	342 (100%)				
Western	329 (74.27%)	317 (75.84%)	393 (100%)			
Eastern	339 (73.38%)	326 (74.42%)	353 (76.41%)	422 (100%)		
Southwestern	271 (66.09%)	267 (70.82%)	282 (68.28%)	281 (63.43%)	302 (100%)	
Southern	337 (67.94%)	324 (68.64%)	355 (72.16%)	375 (74.85%)	293 (63.28%)	454 (100%)

### Additive Partitioning at the Caribbean Basin Scale

**Figure 3:**
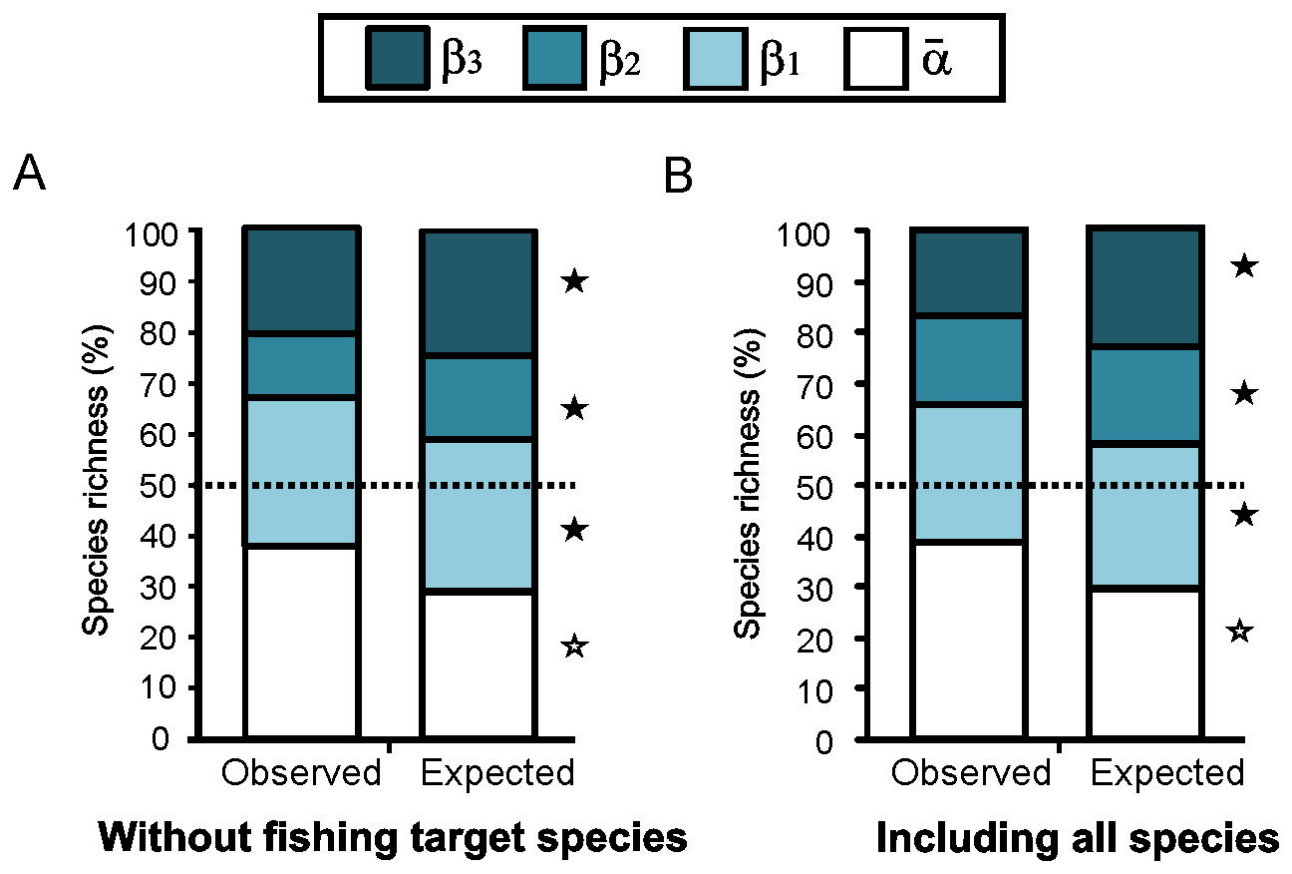
Additive partitioning of reef fish diversity at the Caribbean basin scale. Contributions of average α-diversity and three β-diversity components: β_1_ among sites, β_2_ among subregions, and β_3_ among ecoregions to γ-diversity. A: without fishing target species and B: including all the reef-fish species. The observed partitions (Obs.) are compared with the expected values (Exp.) as predicted by the null model based on 999 randomizations. Results are presented as percentages for easier comparison. Open star symbols: Exp < Obs, p<0.0001. Filled star symbols: Exp > Obs, p<0.0001.

The total γ-diversity at the Caribbean scale was mainly attributed to β-diversity. The diversity percentage explained by all β-components was 61.8%, of which 22.9% (ca.111 species) was among-sites (β_1_), 7.8% (ca. 38 species) among-subregions (β_2_), and 31.1% (ca. 151 species) at the regional level (β_3_). The $\bar{\alpha }$-diversity was higher than expected by chance (p<0.0001) and comprised 38.1% of the total “unfished” species richness at the Caribbean basin spatial scale, ca. 184 average observed species from the total γ-diversity of 484 species (Figure 3A). 

The contribution of β-diversity decreased across hierarchical levels. The observed β-diversity among sites (β_1_) and among ecoregions (β_3_) was lower than expected by chance. Only β-diversity among subregions (β_2_) was higher than expected by chance but it was not statistically significant (p = 0.059). This implies that species variation at this spatial scale is not a consequence of sample design and effort, nor is it merely random. 

### Additive partitioning at the ecoregional scale

$\bar{\alpha }$-diversity explained a very similar proportion of the total γ-diversity for the Bahamian (BE), Eastern (EE), Western (WE), and Greater Antilles (GE) ecoregions with values between 51.4 and 55.2%. The Southwestern ecoregion (SWE) showed the highest $\bar{\alpha }$ contribution (67.7%), whereas in the Southern ecoregion (SE) the β-component (β_1 +_ β_2_) was more important. The partition pattern was very similar for the Bahamian, Eastern, Western, and Greater Antilles ecoregions where the species differentiation among sites (β_1_) was between 32.4% (ca.113 species) and 39.4% (ca. 133 species).

**Figure 4:**
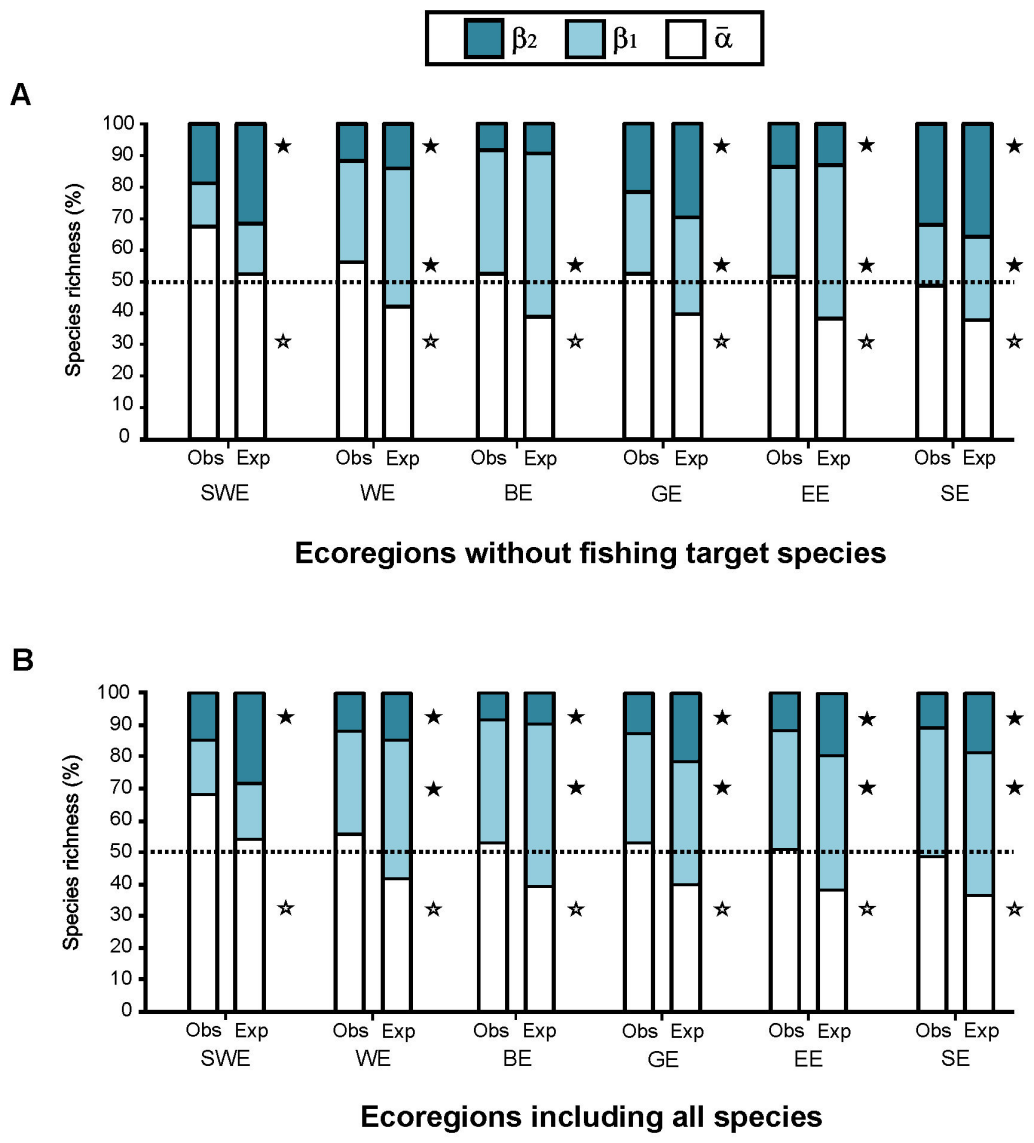
Additive partitioning at the ecoregion level and fishing effects. Contributions of average α-diversity and two β-diversity components: β1 among sites, β2 among subregions, and β3 among ecoregions to γ-diversity. A: without fishing target species and B: including all the reef-fish species. The observed partitions (Obs.) are compared with the expected values (Exp.) as predicted by the null model based on 999 randomizations. Ecoregions abbreviations: Southwestern (SWE), Western (WE), Bahamian (BE), Greater Antilles (GE), Eastern (EE) and Southern (SE). Open star symbols: Exp < Obs, p<0.0001. Filled star symbols: Exp > Obs, p<0.0001. Ecoregions according to Spalding et al. [25].

As was the case for the entire Caribbean basin, the $\bar{\alpha }$-component was greater than expected by chance (p<0.0001) for all partitioning at the ecoregional scale. β-components were in general lower than expected (Figure 4A). Observed β-diversities among sites (β_1_) were lower than expected for all regions except for the Southwestern (p = 0.045). 

### Including fishing target species in the diversity partition

At the Caribbean basin scale, total diversity was explained by β-diversity (61.7%), of which 27.2% (ca.146 species) was among-sites (β_1_), 17.5% (ca.95 species) among-subregions (β_2_), and 17% (ca. 92 species) at the regional level (β_3_). All β-components were lower than expected from a random distribution. $\bar{\alpha }$-diversity was greater than expected by chance (p<0.0001) and comprised 38.3% of the total fish species richness, with ca. 206 average observed species from the total γ-diversity of 539 species (Figure 3B). 

Not all the 55 fishing target species were present in each ecoregion. In the Bahamian ecoregion (BE), 41 fishing target species were found. In the Eastern (EE) and Western (WE) ecoregions the total number of fished species was 46; but they were not the same species. In the Greater Antilles (GE), 35 fishing target species were found. The Southern ecoregion (SE) showed the highest number of species important to fisheries (47) while only 26 were present in the Southwestern region (SWE).

For all ecoregions, the $\bar{\alpha }$-diversity was greater than expected from a random distribution of species (p<0.0001), Figure 4B. Similar to when fishing target species were excluded, the partition pattern was comparable for the BE, EE, WE and GE. In these regions the contribution of lower than expected β_1_ was between 32.4% (ca.127 species) and 38.8% (ca. 147 species) and lower than expected β_2_ was between 8.2 and 12.4% (Figure 4B). 

### Demographic connectivity and diversity partition patterns

**Figure 5:**
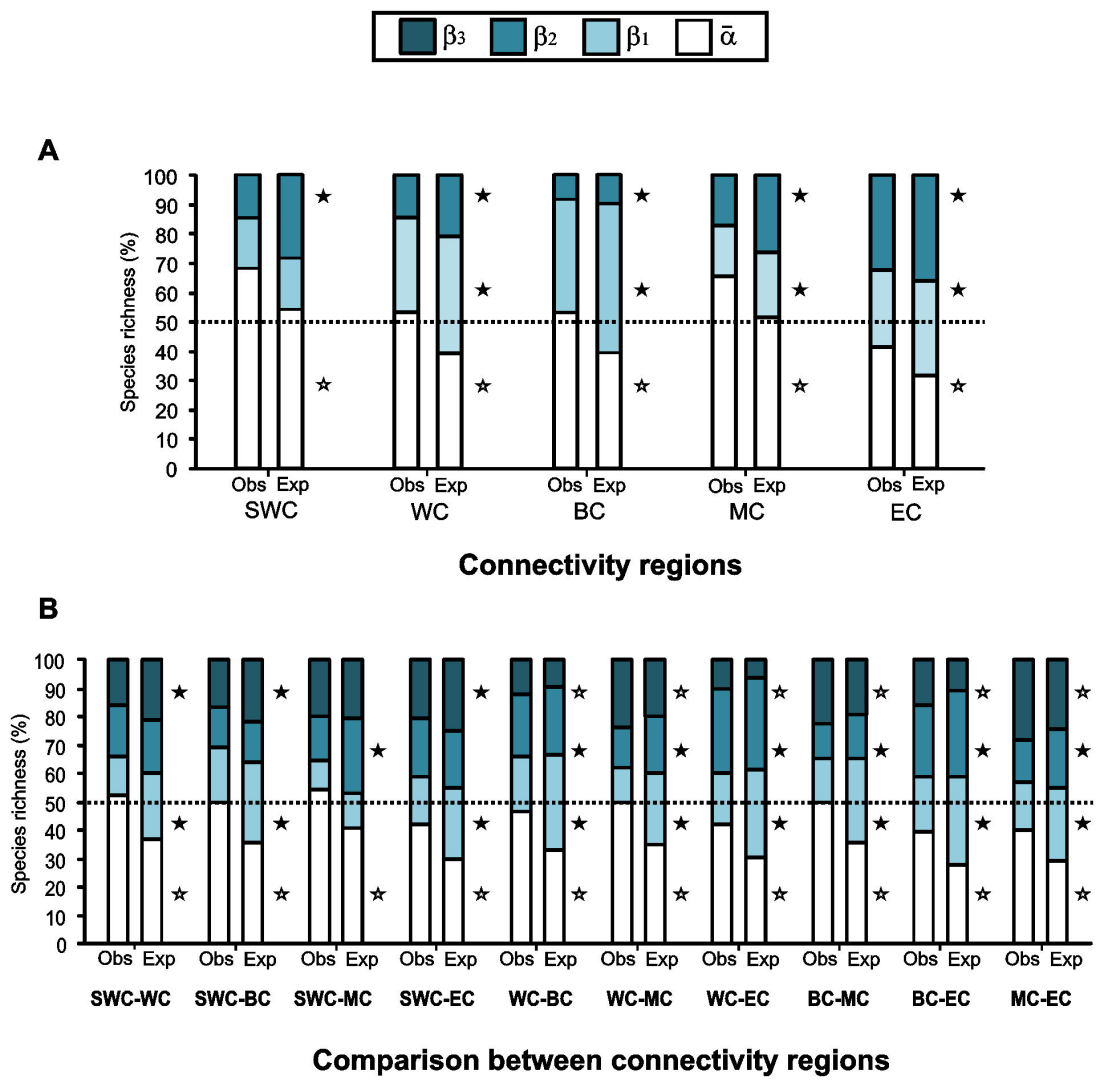
Additive partitioning of reef fish diversity at different scales for the connectivity regions. Contributions of average α-diversity and three β-diversity components: β1 among sites, β2 among subregions, and β3 between regions to γ-diversity. A: partitioning for individual connectivity regions and B: for pairs of regions. The observed partitions (Obs.) are compared with the expected values (Exp.) as predicted by the null model based on 999 randomizations. Connectivity regions abbreviations: Southwestern-Panama Gyre (SWS), Western Caribbean (WC), Bahamas Turks and Caicos (BC), Mixing zone (MC) and Eastern Caribbean (EC). Open star symbols: Exp < Obs, p<0.0001. Filled star symbols: Exp > Obs, p<0.0001. See the text for details on Connectivity regions proposed by Cowen et al. [29].

Considering the connectivity regions proposed by Cowen et al. [33], we found that for all five regions $\bar{\alpha }$-diversity was greater than expected by chance (p<0.0001), and almost all observed β-components were lower than expected (Figure 5A). $\bar{\alpha }$was the most important element for all regions except for the Eastern connectivity region (EC). The contribution of variation among subregions (β_2_) was smaller than or very similar to the contribution among sites (β_1_), except for the EC region.

When comparing between connectivity regions $\bar{\alpha }$-diversity was also higher than expected from a random distribution (p<0.0001). β_3_ was higher than expected for all comparisons except when SWC was included (Figure 5B). In general β-components were low within connectivity regions and high when comparing between them (Figure 5). The partition between SWC-WC and SWC-MC regions showed the lowest observed β-component.

## Discussion

### General richness pattern and species composition

The known species richness of Caribbean reef fishes is between 500 and 700 species [53,63]. After the references-based geographic distribution corroboration, we used 539 species for the analysis, which seems to be a fair representation of the total documented richness of reef fish species for the Caribbean.

Region-scale diversity assemblages were in general homogenous, primarily reflecting a similar species composition with differentiation in the southern Caribbean. The relatively small number of singleton species, the high percentage of similarity within sites and regions and the very similar values of the Russel and Rao coefficient among regions suggest that there is an important number of species that are common to many sites throughout the Caribbean. However, there is some variation in species composition between the different spatial scales. The combined PERMANOVA and PERMDISP allowed us to detect that variation in composition was highest among the studied sites. In particular, the strongest differentiation was between the sites of the Southern ecoregion (high degree of dispersion in the NMDS plot). We think this differentiation could be related to the fact that sites in the Southern ecoregion are located in very different physicochemical provinces [64]. Trinidad and Tobago reefs are influenced by freshwater from the Orinoco River, an area characterized by turbid waters and low salinity [64]. Aruba, Curacao, Bonaire and some Venezuelan reef sites are found in relatively clear and warm waters of high salinity, while other Venezuelan reef sites are influenced by wind-driven upwelling near the coast [64,65]. 

### Additive partition, more than random sampling

The average α-diversity was higher than expected in all analyses. This pattern was geographically consistent for the ecoregions, suggesting that fish assemblages within ecoregions are more homogenous than expected. One explanation for this important finding could be related to the evolutionary history of the area. The Caribbean today seems to possess only a portion of an old and possibly widespread fish fauna [66]. Species loss has occurred in the past [67], with only those species able to resist intense environmental pressure surviving in many places. 

Another explanation for the high observed $\bar{\alpha }$-diversity could be related to a ubiquitous larval pool or high demographic connectivity among regions. Movements between spatial units (i.e. dispersal) would act as a homogenizing force, raising the $\bar{\alpha }$-diversity and reducing the β-component [68]. This would allow a large number of species to be common to all studied regions. β-components were lower than expected by chance in all ecoregions, indicating that fish species within each ecoregion are a subsample of the same species pool. The connectivity of reef fishes is typically accomplished by the dispersal of pelagic larvae [32]. The biogeography and oceanography of the Caribbean show a strong potential for demographic connectivity due to its stepping-stone geography and a total distance along a current track of more than 4000 km [69]. We analyze whether the demographic connectivity is driving the α- and β-diversity patterns later in this section.

### The spatial scale associated with the highest β-diversity

β-diversity reflects the importance of variation at each scale, as it contributes to the total diversity of a system [70]. For coral reef ecosystems off the eastern coast of the Yucatan Peninsula, Rodríguez-Zaragoza and Arias-González [23] found that β-diversity was the most important component at all the studied spatial scales; they found it to be higher between sites. At the Caribbean scale we found that the β-components explained nearly 62% of the total diversity. Although it was less than expected, we found the greatest species differentiation among sites. Our results suggest that the site spatial scale within regions is a very important level for conserving the total diversity of Caribbean coral reef fishes; this finding partially corroborates that of Semmens et al. [62], namely that conserving diverse sites regardless of region is effectively equal to conserving diverse sites within each region. We also found that observed β-components decreased across hierarchical levels. In a recent revision, Holland [70] found that β-diversity increased across hierarchical levels in just half of the 15 studies reviewed. Thus, there is apparently no simple rule for the size of β-diversity at progressively larger scales [70]. 

It is important to consider that we included information from protected (e.g. marine reserves) and non-protected sites. This difference in protection could be a factor increasing β-diversity at the site scale. Species diversity tends to be higher inside marine protected areas [28,71]. Habitat differences in coral cover and complexity [72,73] as well as variation in depth [72] or wave exposure [74] could generate differentiation in species composition among sites. Other factors such as pollution [75], coral-reef phase shift [76] and fisheries could also affect β-diversity [28].

### Additive Partitioning and Fisheries Effects

The direct effects of fishing could increase, decrease, or have no effect on species richness or distribution, depending on the relative impact on common versus rare species [28]. Observed distributions with and without fisheries target species were very similar in terms of proportions. This finding suggests that our fishing scenario cannot explain the fish diversity partition pattern observed at the Caribbean basin scale. 

When comparing the diversity distributions with and without fishing target species at the ecoregional scale, the dominance of the β-diversity components changed for the Southern and Southwestern regions. In the Southern ecoregion some fishing target species were common to all sites but many others were common to just two or three sites of the same subregion. Therefore, the Southern ecoregion without fishing target species showed more similar sites in terms of species composition but with greater differences between subregions. We think this could be related to environmental differences among site locations, with the Southern reefs located in very different physicochemical provinces as previously mentioned.

Excluding the fishing target species and using presence-absence data are a coarse way of investigating the fisheries effect on diversity patterns; some species could be less abundant or diminish in size as a consequence of fisheries before becoming completely absent. However, our fishing scenario shows the potential loss of local species to be less than that caused by other kinds of disturbances on coral reef systems, e.g. coral cover reduction. During experimentally induced disturbances, in which coral cover was reduced by 16-36%, the proportional decline in fish diversity was 1.8-2.3 times the proportional coral loss [77]. 

Local strategies of fish species usage should also be taken into account in order to obtain more realistic results. The importance of certain target species varies with location. For example, parrotfishes are an important food source in some countries [78], while in others these species are not highly commercial [79]. 

We found a more heterogeneous distribution of the reef-fish species and the fishing target species along the northern coast of South America. The ecological consequences of such a difference are hard to predict. Fisheries exploitation affects not only target stocks but also communities of organisms, ecological processes and entire ecosystems [80]. Reef fish may perform different functional roles and can have different effects on the diversity and abundance of prey species [37]. The loss of a species and its functional roles can lead to a reduction in ecological stability [81,82]. The ecosystem can become less resilient to perturbations such as hurricanes or invasions [6,83]. 

### Demographic connectivity and diversity partition patterns

We expected to find high β-diversity among weakly connected areas and lower β-diversity among highly connected locations. β-components were low within regions proposed by Cowen et al. [33] and significantly higher when comparing between them (β_3_), showing a more similar species composition among its highly connected subregions and sites. This has very important conservation implications since high connectivity can buffer populations from local extinction and facilitates post-disturbance recovery [69]. 

Within connectivity regions the highest β-diversity was at the site scale and observed $\bar{\alpha }$-diversity was higher than expected in all partitioning analyses; fish assemblages throughout the Caribbean are more homogenous than expected, as we found with the Spalding et al. [25] bioregionalization. Some studies suggest a basin-scale mixing among reef fish populations across the Caribbean [84,85], but as we have seen, relative variation in species composition between regions persists. 

We detected higher than expected β-diversity between some suggested weakly connected regions such as distant BC and EC. In general, the difference between connectivity regions (β_3_) was higher than expected (from a random distribution) for seven of ten pair-wise comparisons, showing that there are differences in composition between these regions. 

According to Cowen et al. [33] the Bahamas and the Turks and Caicos Islands are largely isolated from the rest of the Caribbean, forming an enclave of high connectivity among its islands. We found that sites in this region were similar in terms of species composition with lower than expected β_1_, suggesting there is an important intra-connection. 

Other regions such as the SWC (similar in terms of geographic delimitation as SWE) have also been described as highly isolated [33]; this was supported by an important number of unshared species with other regions and a higher than expected β_3_ between SWC and EC. These two regions have been described as moderately isolated from each other along a meridional break that goes from the western end of Puerto Rico south to Aruba (Figure 1). Its relative isolation has been related to a high proportion of self-recruitment partially due to low importation from upstream locations and the proximity to the semipermanent Panama-Colombia Gyre [33]. Is important to consider that the high $\bar{\alpha }$-diversity of SWC might be explained by the reduced number of sites.

The Eastern connectivity region (EC), broadly composed by the Eastern and Southern ecoregions, showed a differential pattern in its β-component and was the only connectivity region where β-diversity was more important. The explanation for this is related to large differences in composition between sites along the northern South American coast in SE, which are located in very different physicochemical provinces. 

The connectivity regionalization proposed by Cowen et al. [33] partially explained the similarity within regions and differences between them. However, it is clear that the Caribbean reef-fish diversity pattern is not only driven by present-day connectivity but also has important biogeographic and evolutionary components. Contemporary distribution patterns reflect biological and physical processes operating at multiple spatial scales, over both evolutionary and ecological time scales [69]. Biogeographic and evolutionary mechanisms seem to determine the species distribution in the different ecoregions. Limited connectivity could be responsible for demographic isolation in some areas with an important number of unshared species. Each regionalization was based on different processes but the results using ecoregions and connectivity regions were complementary. Ecoregions are based on a broader assemblage of processes such as taxonomic configurations, influenced by evolutionary history, patterns of dispersal, and isolation [25]; whereas connectivity predictions depend greatly on the assumptions made concerning larval behavior, the accuracy of dispersal and the hydrodynamic model used [32]. Ecoregions allowed us to detect changes in β-diversity throughout the Caribbean, while connectivity explained part of this compositional variation. 

### Final comment

Our study helps to improve the understanding of diversity partitioning at different scales, and some of the effects that fisheries and connectivity might have. 

To the best of our knowledge, this is the first study to use additive partitioning to analyze the diversity pattern of coral reef fish at the Caribbean basin scale. This approach helped us to identify that the β-component explains nearly 62% of the total diversity of Caribbean coral reef-fish and that observed $\bar{\alpha }$-diversity was higher than expected at all spatial scales and for both types of regions. The demographic connectivity partially explained the diversity distribution pattern we have found. Other studies indicate that geographic or physical constraints as well as connectivity [33,86] seem to play an important role in shaping fish species communities. Paleontological, phylogenetic and biogeographic data strongly suggest that present-day diversity patterns have an important historical component [87,88].

Further studies could include the separation of species by guilds or by commercial importance, as Sandin et al. [89] already started to explore, in order to try to identify changes in diversity patterns. We also need to address how much of the diversity partitioning is explained by habitat differences, e.g. reef condition, coral cover, benthic structure or complexity. 

## Supporting Information

Table S1 **Scientific name, families, order and REEF data base code of all fish species used in this study.**
(XLSX)

Table S2 **List of the species common to all regions and the species that appeared in only one region.**
(XLSX)
